# The Lister Reconstruction: A Novel Technique to Reconstruct the Patellar Tendon

**DOI:** 10.7759/cureus.99935

**Published:** 2025-12-23

**Authors:** Ali Al-Kulabi, Asad Ali, Pranav Mishra, Maheshwaran Logeshwaran, Satish Babu, Raj Thakrar

**Affiliations:** 1 Orthopaedics, East and North Hertfordshire NHS Foundation Trust, Stevenage, GBR

**Keywords:** autograft, novel technique, patellar tendon, reconstruction, trauma

## Abstract

Chronic patellar tendon ruptures are uncommon but debilitating injuries that present a significant reconstructive challenge, particularly in the context of pre-existing tendinopathy. Direct repair is frequently not feasible due to tendon retraction, poor tissue quality, and proximal patellar migration, necessitating augmentation or reconstruction techniques.

We report a novel method of patellar tendon reconstruction in a 27-year-old male with a chronic rupture and underlying tendinopathy. The technique combines direct repair with hamstring autograft augmentation, utilizing an adjustable suspensory loop device for proximal fixation at the inferior pole of the patella and a transosseous tibial tunnel with interference screw fixation distally. This approach restores the native vector of the patellar tendon while permitting intraoperative adjustment of patellar height. After harvest of the gracilis and semitendinosus autografts, tunnels were prepared in the patella under fluoroscopic guidance. Adjustable cortical fixation was employed proximally with docking of the graft at the inferior pole, while distal fixation was achieved through a tibial tunnel. The residual native tendon was repaired with Krakow sutures, supplemented by autograft augmentation, and reinforced with retinacular closure. Intraoperative imaging confirmed restoration of patellar height and alignment.

The patient was mobilized with a range-of-motion brace and partial restrictions. Intra-operative radiographs confirmed maintenance of patellar height, with satisfactory functional recovery and no evidence of graft failure.

To our knowledge, this is the first reported case of patellar tendon reconstruction combining direct tendon repair with hamstring autograft augmentation using adjustable suspensory loop fixation proximally and transosseous tibial tunnel fixation distally. This construct provides both biological reinforcement and biomechanical stability, while restoring the native pull vector of the extensor mechanism. Further evaluation in larger series is warranted to determine long-term functional outcomes and graft incorporation.

## Introduction

Chronic patellar tendon ruptures are rare but serious injuries that present a significant reconstructive challenge to the treating physician [1]. They often present due to delayed diagnosis, neglected or missed acute ruptures, resulting in proximal patellar migration, quadriceps shortening, tendon retraction, and poor tissue quality [1,2].

Unlike acute cases, which may be amenable to direct repair, chronic ruptures typically exclude primary end-to-end approximation and are associated with marked functional deficit [3].

The extensor mechanism is critical to the function of the lower extremity. It is responsible for extending the knee and resisting knee flexion - a crucial aspect of standing with a flexed knee and, therefore, ambulation [4].

Given these factors, surgical management generally necessitates augmentation or reconstruction with autograft, allograft, or synthetic substitutes to restore the extensor mechanism and optimize patient outcomes [3,5].

The objective of this case report is to describe a novel technique for the reconstruction of the patellar tendon using a hamstring tendon graft.

## Case presentation

Preoperative imaging

**Figure 1:**
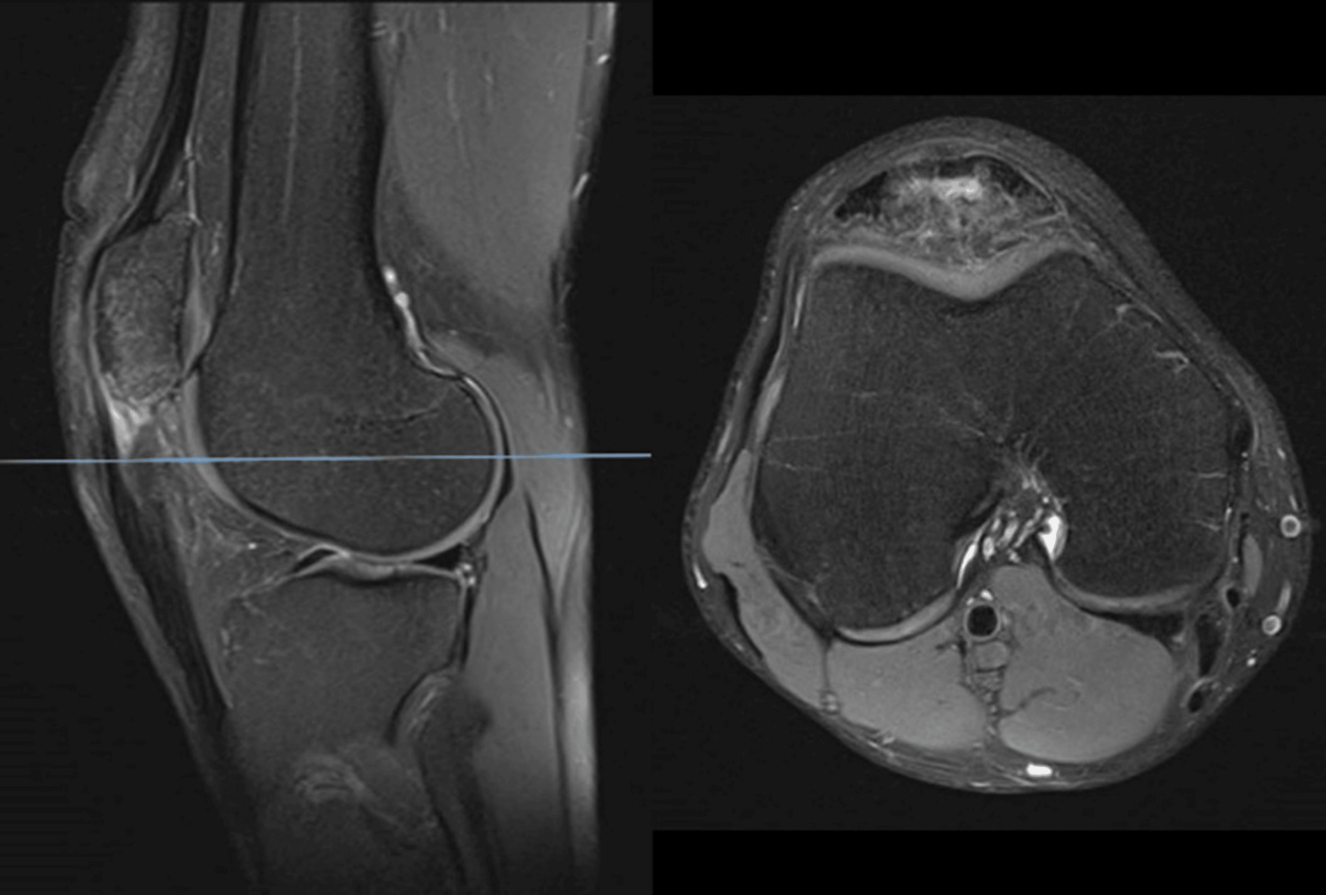
Sagittal and axial T2-weighted MRI images at first presentation, demonstrating intact extensor mechanism function. The proximal patellar tendon shows intact fibers with high signal intensity consistent with tendinopathy. MRI, magnetic resonance imaging

**Figure 2:**
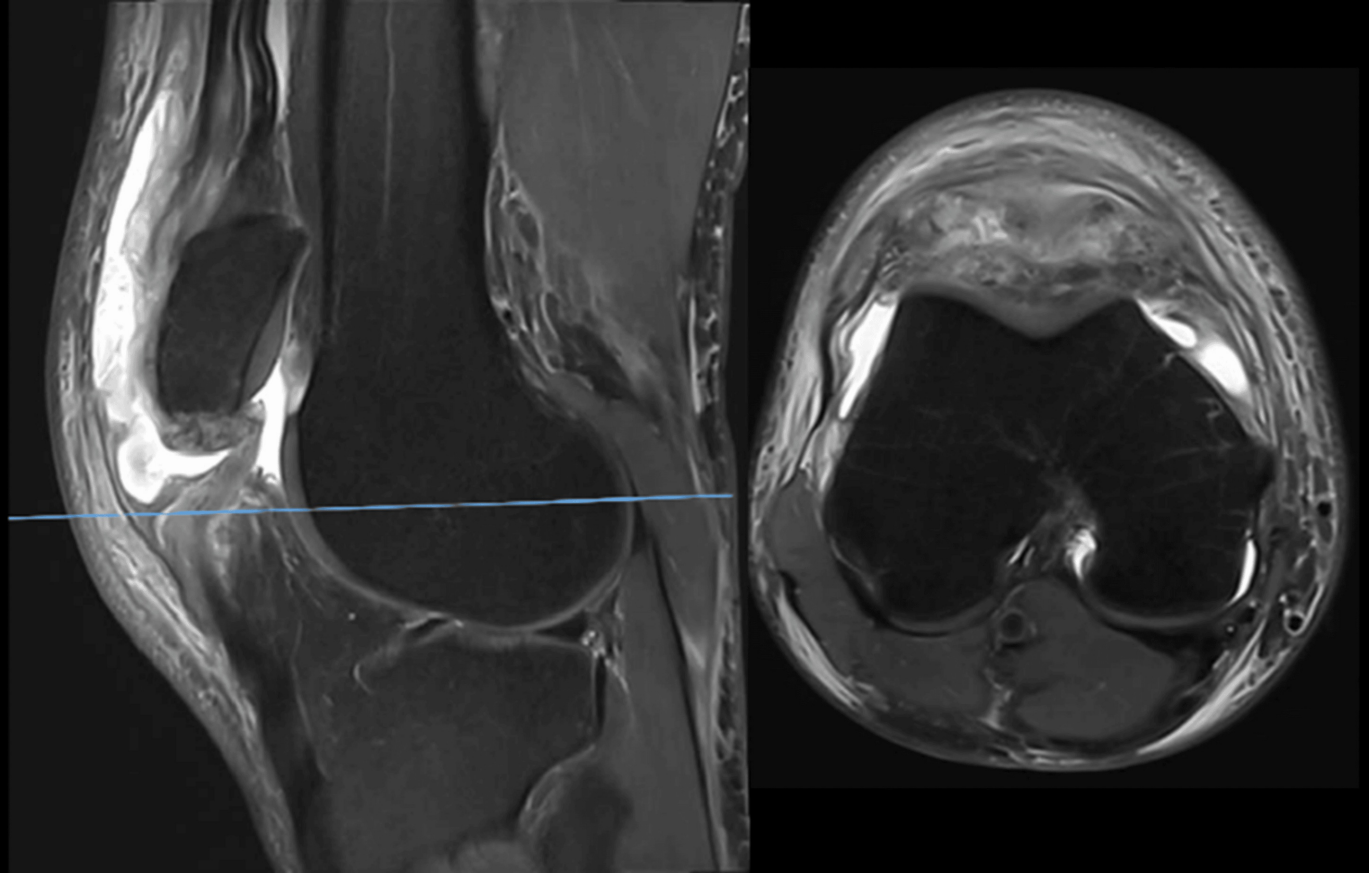
Sagittal and axial T2-weighted MRI images at second presentation, demonstrating deficient extensor mechanism function. The proximal patellar tendon fibers are discontinuous, consistent with rupture. MRI, magnetic resonance imaging

A young, fit, and healthy 27-year-old male presented to our service with acute knee swelling after sustaining an injury playing football. He previously had an injury to the same knee, subsequent to which he developed instability. Magnetic resonance imaging (MRI) performed at that time demonstrated patellar tendinopathy. He subsequently underwent a course of physical therapy. He later sustained another injury while playing football and presented to our service one year later. On examination, he was unable to perform an active straight-leg raise and was tender to palpation at the distal pole of the patella. The active range of motion was limited to 10-15 degrees of flexion. A repeat MRI demonstrated a complete rupture of the patellar tendon, as shown in Figures 1-2. Treatment options were discussed with the patient, and given a delay in presentation of approximately two months, a reconstructive procedure was considered appropriate.

Operative method

Approach

Care was taken to prepare and drape the lower limb as proximally as possible to allow for adequate extension should a quadriceps release or V-Y plasty have been required. A midline incision was made over the patella, exposing the patellar tendon, with sufficient proximal extension to visualise the tendon proximal to the superior pole of the patella.

The paratenon was elevated as a separate layer to facilitate closure.

**Figure 3:**
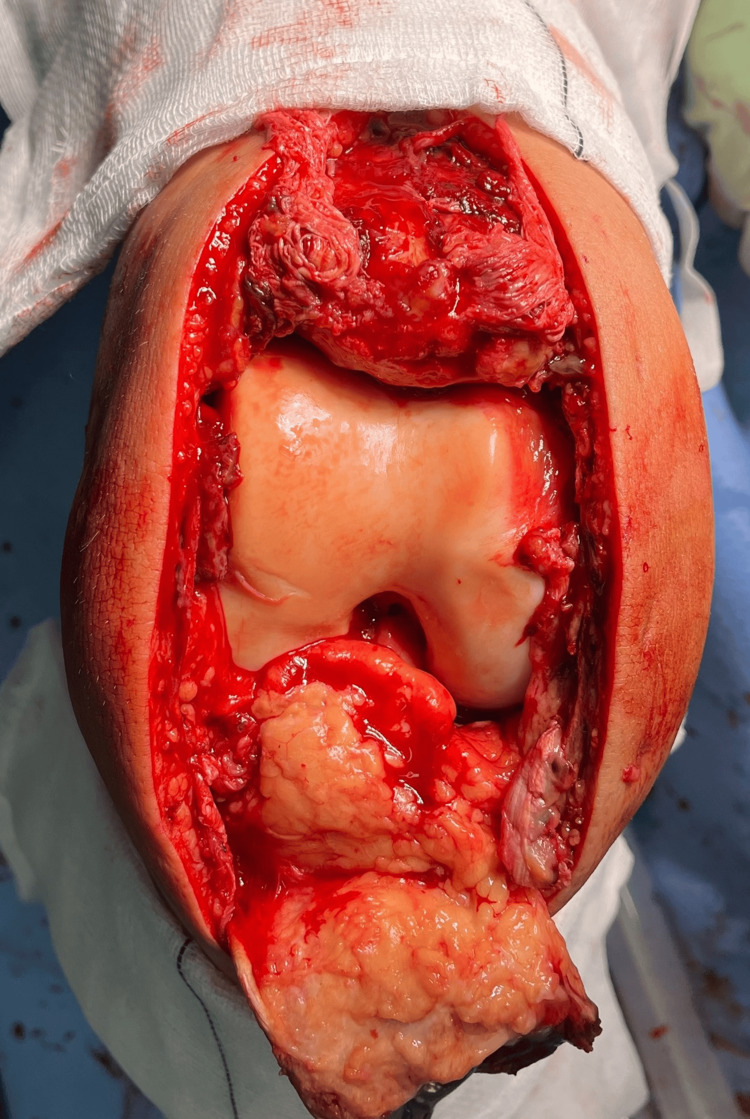
Frontal view of the knee. From superior to inferior: patella, femoral cartilage, fat pad, and patellar tendon hanging anteriorly (note its tendinopathic appearance).

The intra-articular haematoma was evacuated, and the patellar tendon defect was visualised. The patellar tendon was dissected proximally from the retinaculum and fat pad. Figure 3 demonstrates the appearance at this stage. The inferior aspect of the patella was debrided centrally in the sagittal plane to ensure appropriate docking sites.

Tendon Harvest

The sartorial fascia was exposed through the midline incision using palpation of the hamstrings, known anatomical landmarks, and by identifying and following the sentinel vessel [6]. The fascia was incised, and the distal attachments of the gracilis and semitendinosus were identified. A tendon stripper was used to harvest both tendons, preserving the distal attachments at the pes anserinus, and a #2 FibreLoop was whip-stitched to the free proximal ends. The length of each harvested tendon was measured, with a minimum of 260 mm, and doubling the grafts at their midpoints produced a diameter of approximately 7-9 mm.

Patellar Preparation

A 2.4 mm anterior cruciate ligament (ACL) islet guidewire was passed under image intensifier (II) guidance into the centre of the inferior pole of the patella toward the superior pole. Two additional wires were passed medial and lateral to the central wire, parallel to it. The wires were monitored on anteroposterior (AP) and lateral views to ensure they were not too superficial and did not breach the articular surface, aiming to remain subchondral with sufficient space for a 7-9 mm diameter socket for the graft. The wires were brought out through the quadriceps tendon as close as possible to the superior pole of the patella. Techniques may vary, but using a Langenbeck retractor to push the quadriceps tendon posteriorly allowed a safe route for the islet wires and protected the skin.

The central wire was then over-drilled with a 4.5 mm drill to allow passage of an Arthrex TightRope® Reverse Tension (TR-RT; Arthrex Inc., Naples, FL) device along its entire length. The distal 25 mm was drilled with a 7-9 mm acorn reamer to create a docking site for the hamstring graft.

Native Patellar Tendon Reattachment

The proximal patellar tendon was prepared by running two Krakow sutures with #5 FiberWire, leaving four free ends at the proximal tendon. The free ends of the sutures were passed through the islet guide wires and the patellar tunnel, exiting at the superior pole of the patella. In our experience with this technique, methods of passing can include: (1) passing both free ends of each pair through their corresponding medial and lateral patellar tunnels, or (2) passing a single strand through the corresponding medial and lateral tunnels and the central strand of each pair through the central tunnel using a loop of polydioxanone (PDS) for later shuttling of the TR-RT.

Initial tensioning of the Krakow FiberWire sutures was performed by throwing the first double throw and holding with an artery clip using one of the following techniques: (1) medial-to-lateral pairs, or (2) the central strand with its corresponding medial and lateral strands. This revealed the remaining defect and demonstrated why primary repair was not recommended. In our experience, tightening the primary Krakow sutures to close the defect and achieve tendon-bone contact placed undue stress on the tendon, causing damage and resulting in patella baja, which could be seen under II.

Graft Tendon Preparation and Attachment

**Figure 4:**
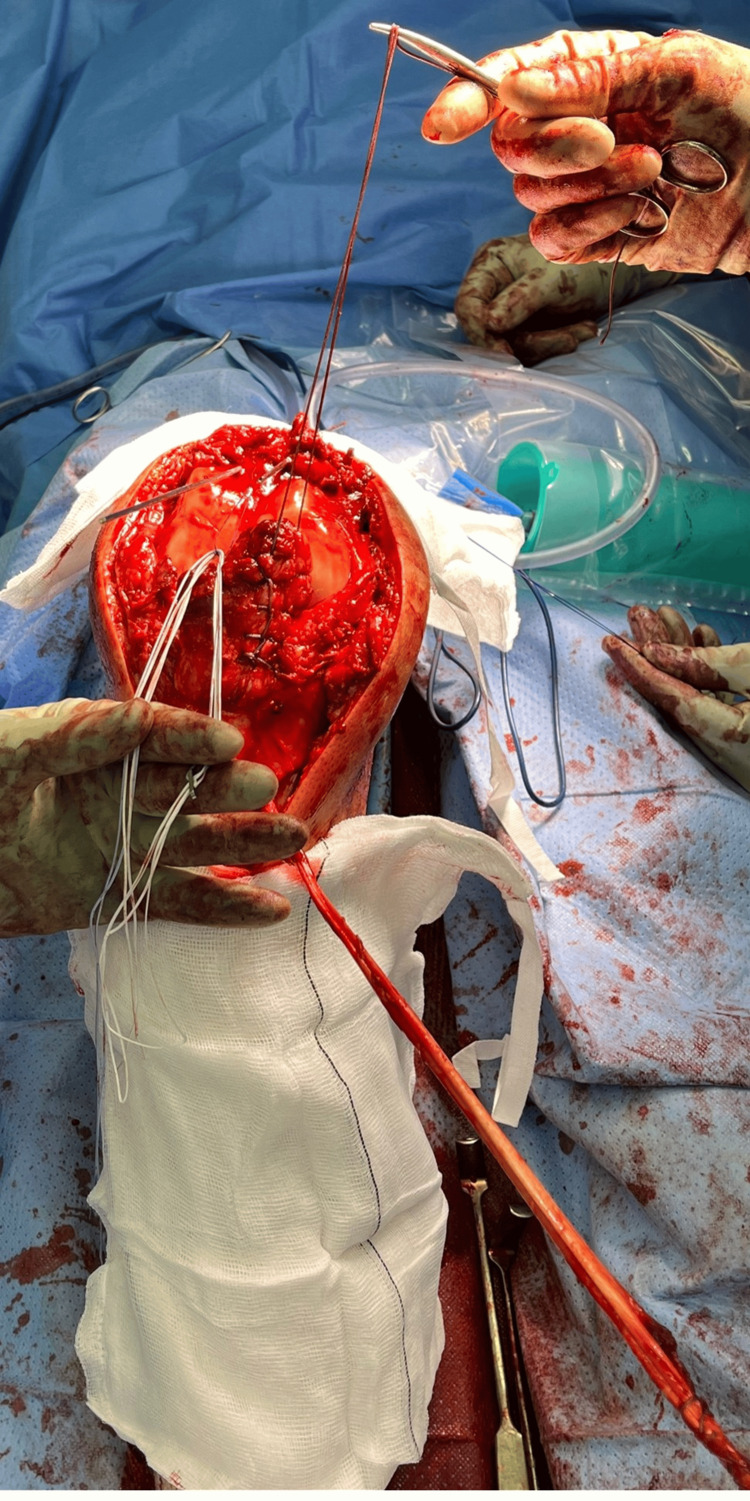
Suture preparation, shuttling, and docking. The patella has medial and lateral ACL islet guide wires. The central tunnel contains a loop of PDS, ready to shuttle the Arthrex TR-RT (white and blue sutures displayed at six and seven o’clock). The patellar tendon has a medial Krakow stitch (displayed at one o’clock), awaiting the lateral Krakow stitch. The hamstring autograft was harvested and whip-stitched with Arthrex #5 FiberLoop (displayed at four o’clock). TR-RT, TightRope-reverse tension; ACL, anterior cruciate ligament; PDS, polydioxanone

The patella was reduced to the desired height using a pointed reduction forceps and checked on II. The medial limb of the hamstring graft was laid over the inferior central patella, and 25 mm was measured and marked. The adjustable suture loop of the Arthrex TR-RT was passed around the hamstring tendon graft at this point. Size 1 Vicryl suture was used to tie the double strand together at its patella docking site, approximately 2 cm from the tip. The loop PDS was used as a shuttle to pass the free end of the TR-RT through the central patella tunnel (Figure 4). The TR-RT button was then passed through the tunnel and flipped onto the cortex of the superior pole of the patella under the quadriceps tendon and checked on II. The knee was flexed to 30 degrees, and the graft was docked into the socket. Final sinking of the TR-RT was then performed.

The Krakow stitches were then finally tied in the pairs outlined above. In our experience with this technique, they can be placed either on top of the quadriceps tendon or beneath it so that the sutures rest on the superior pole of the patella. To achieve the latter, the sutures must be diverted through their quadriceps slits, usually retrieved from the central slit using an artery clip.

**Figure 5:**
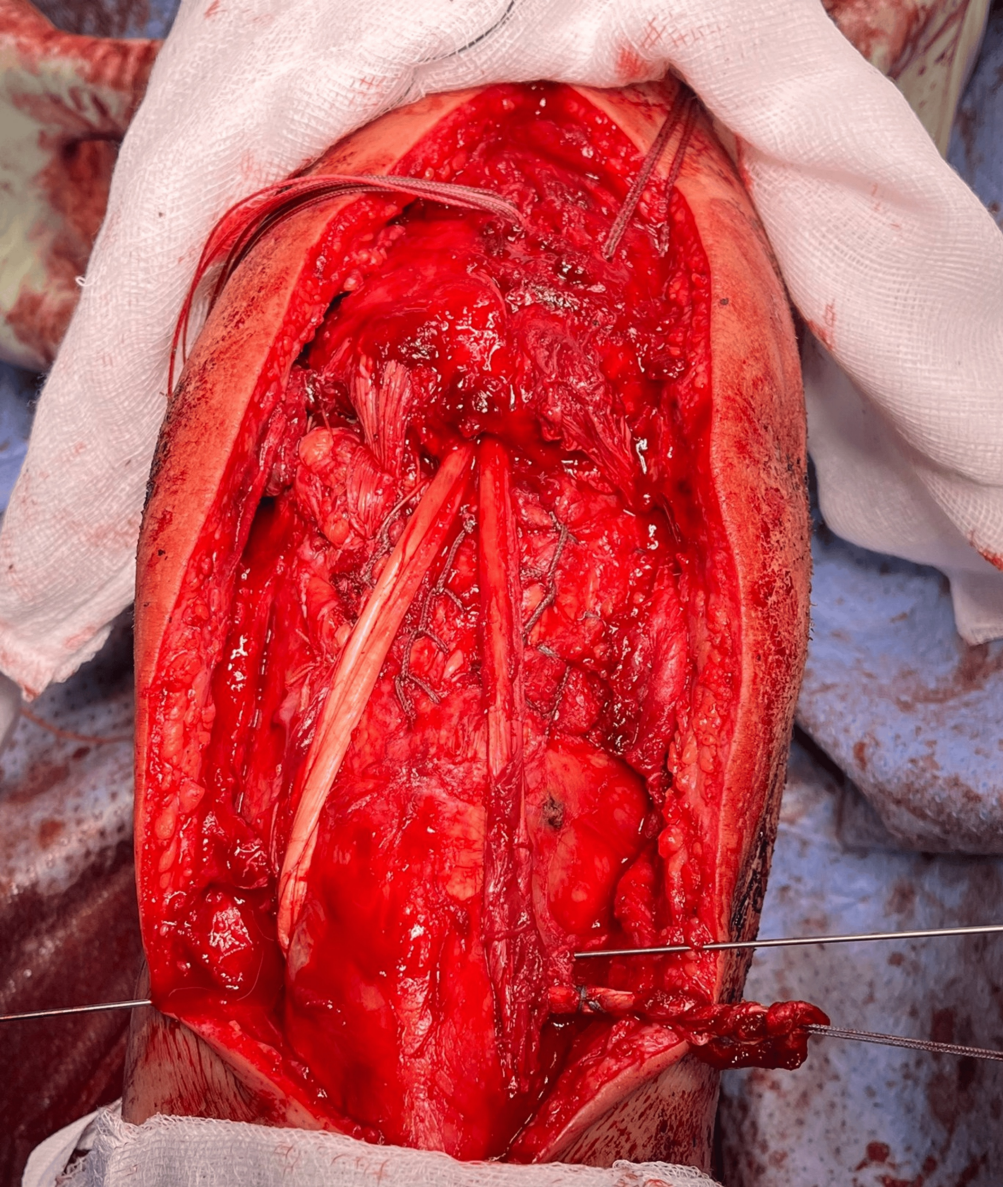
The native patellar tendon bed is displayed with a Krakow stitch tied on the superior pole of the patella, forming one bundle of four sutures at the one o’clock position. The Arthrex TR-RT sutures are displayed at the superior pole of the patella at the nine o’clock position. The medial limb of the hamstring autograft runs from the pes anserinus to the patella, and the lateral limb courses back down to the lateral proximal tibia and through the tunnel (displayed with an ACL islet guide), ready for final fixation. TR-RT, TightRope-reverse tension; ACL, anterior cruciate ligament

With the knee maintained at 30 degrees of flexion, a transverse 7-9 mm tunnel was drilled in the lateral proximal tibia, proximal to the pes anserinus, to prevent clashing with the insertion site. The free end of the hamstring graft was passed into this tunnel using an islet guide wire (Figure 5) and secured with an Arthrex FastThread Polyether ether ketone (PEEK) interference screw, one size larger than the tunnel diameter. Any remaining graft was then trimmed.

**Figure 6:**
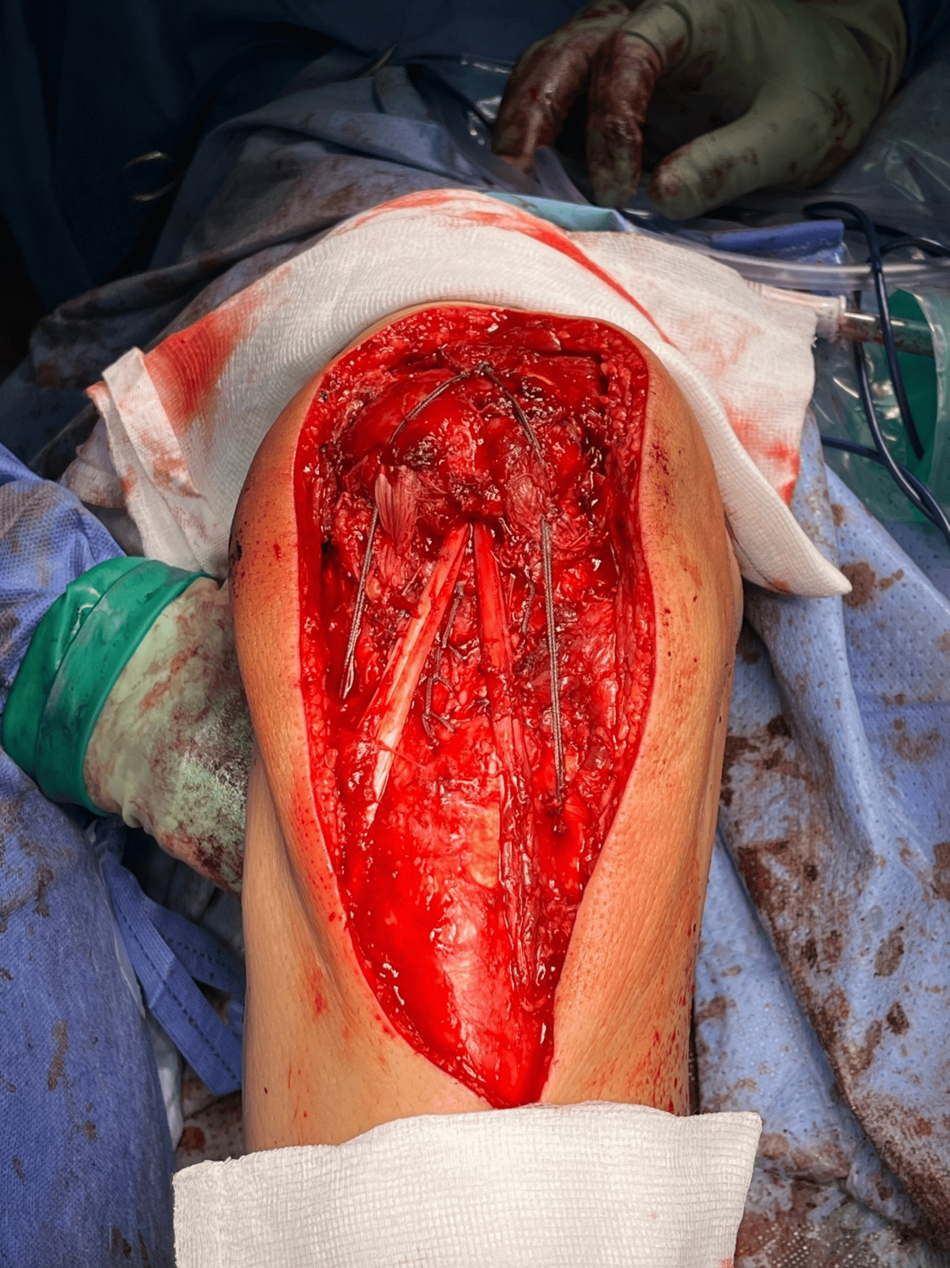
The final Lister patella reconstruction. Meticulous medial and lateral retinacular closure was performed. The free ends of the Krakow stitch were brought down in pairs on either side of the proximal tibia and secured 2 cm below the joint line with an Arthrex 4.75 mm SwiveLock® (Arthrex Inc., Naples, FL), acting as de-tensioning sutures at 30 degrees of knee flexion.

Meticulous medial and lateral retinacular closure was performed. The free ends of the Krakow stitch were brought down in pairs on either side of the proximal tibia and secured 2 cm below the joint line with an Arthrex 4.75 mm SwiveLock (Arthrex Inc., Naples, FL), acting as de-tensioning sutures at 30 degrees of knee flexion. The final reconstruction was then completed (Figure 6).

Follow-up

The patient was placed in a range-of-motion knee brace. Full weight-bearing was allowed through the leg, but the brace had to be locked in extension for six weeks. The range of motion permitted was 0-45 degrees from week 0 to week 3, 0-90 degrees from week 3 to week 6, and free range of motion thereafter. X-rays were obtained at six weeks and three months to assess patella height at 30 degrees of knee flexion.

## Discussion

Patellar tendon rupture in the context of pre-existing tendinopathy presents unique challenges, as the degenerative tendon is often unsuitable for reliable direct repair alone. Augmentation techniques using autografts together with tendon repair have been shown to reduce re-rupture risk and enhance repair strength [7-9].

Several techniques are available for patellar tendon reconstruction, but no gold standard has been established. Techniques vary by the type of graft used, surgical approach, proximal fixation method, and distal fixation method [7].

The patellar tendon reconstruction technique we report uses an open surgical approach with a hamstring autograft, proximal fixation using an adjustable loop device, and distal fixation via a transosseous tibial tunnel, augmented by patellar tendon debridement and repair using whipstitch sutures docked in two additional patellar tunnels medial and lateral to the central tibial tunnel.

Graft options for patellar tendon augmentation include autograft and allograft. Autografts are more biocompatible and have been shown to result in stronger fixation and faster incorporation compared to allografts of comparable size and nature [8]. However, autografts do result in harvest site morbidity. The use of allografts for patellar tendon reconstruction has been reported before. Hamstring autograft was used in this case as the risk and benefit of hamstring autograft is well established and is acceptable in our opinion. Furthermore, morbidity is reduced because the graft is harvested through the same surgical incision [8].

Different patellar tendon augmentation graft fixation methods have been described before. Our use of an adjustable suspensory loop device for proximal fixation in this case provides strong cortical fixation with the added advantage of adjustable tensioning, commonly utilized in ligament reconstructions such as ACL reconstruction [10]. Its application in patellar tendon reconstruction allows fixation at the biomechanically native inferior pole of the patella, re-establishing the normal pull vector while also allowing secure intraoperative adjustment of patella height. This technique provides both biological reinforcement and mechanical stability. This is in comparison to other techniques that are proximally docked to the patella at the proximal or mid-body, with ascending and descending graft limbs at the sides. This requires a longer graft and results in asymmetric graft tensioning. Furthermore, it is biomechanically inferior as it does not recreate the native point of pull at the inferior pole of the patella [11]. Other techniques using a fixed loop device and suture anchors at the inferior pole of the patella have been described, but it lacks the ability to adjust the tensioning of the patellar tendon and patella height. Furthermore, in the case of arthroscopic patellar tendon reconstruction, the minimally invasive surgical approach precludes the ability of repairing the ruptured tendon and extensor mechanism. Augmentation of the patellar tendon without any repair may result in a weaker and less robust construct.

The distal fixation of the patellar tendon reconstruction was done using a transosseous tunnel posterior to the tibial tubercle using an interference screw. This fixation method has been previously reported with a low risk of failures [7-9,11].

The remnants of the patellar tendon and extensor mechanism were repaired as described previously. This was done to increase the strength of the repair construct.

To our knowledge, this is the first reported case of patellar tendon reconstruction combining direct repair with hamstring autograft augmentation using an adjustable suspensory loop device fixation proximally and transosseous tibial tunnel fixation distally for patellar tendon rupture in the setting of chronic tendinopathy. While short-term intraoperative stability was achieved, further follow-up is required to assess long-term outcomes, including graft incorporation, return to function, and re-rupture risk. If validated in larger series, this method could provide a reproducible and biomechanically advantageous approach for managing complex patellar tendon ruptures.

At present, the primary limitation of this study is the lack of high-quality, high-volume data to support the theoretical improvement in long term graft success, particularly in high-functional-demand patients. We plan to collect further data on the postoperative functional outcomes of the Lister technique.

## Conclusions

This case highlights several important learning points. Repairing a patellar tendon rupture in tendinopathic tissue poses a significant challenge, as standard end-to-end techniques are often ineffective due to the compromised quality of the native tendon. Incorporating hamstring autograft augmentation provides both biological reinforcement and biomechanical strength, offering a more robust construct than direct repair alone. The use of adjustable suspensory loop devices facilitates secure fixation at the inferior pole of the patella while allowing intraoperative adjustment of graft tension and restoration of patellar height. Distally, fixation through a transosseous tibial tunnel has proven to be a reliable method, providing stable anchorage of the graft. When combined with meticulous extensor mechanism repair, this augmentation technique delivers sufficient intraoperative strength and stability. Ultimately, this novel reconstructive approach restored the native pull vector of the patellar tendon, allowing the graft to withstand greater force and potentially reducing the risk of re-rupture in patients with high functional demands. Nevertheless, further studies with long-term follow-up are required to assess the durability of the repair and functional outcomes over time.
